# Developing Core Outcome Sets (COS) and Core Outcome Measures Sets (COMS) in Cosmetic Gynecological Interventions: Protocol for a Development and Usability Study

**DOI:** 10.2196/28032

**Published:** 2021-11-15

**Authors:** Stergios K Doumouchtsis, Vivek Nama, Gabriele Falconi, Maria Patricia Rada, Jittima Manonai, George Iancu, Jorge Milhem Haddad, Cornelia Betschart

**Affiliations:** 1 Department of Obstetrics and Gynecology Epsom & St Helier University Hospital NHS Trust London United Kingdom; 2 Laboratory of Experimental Surgery and Surgical Research N.S. Christeas Athens Greece; 3 Institute of Medical and Biomedical Education St George's University of London London United Kingdom; 4 School of Medicine American University of the Caribbean Coral Gables, FL United States; 5 School of Medicine Ross University Miramar, FL United States; 6 CHORUS: An International Collaboration for Harmonising Outcomes, Research and Standards in Urogynaecology and Women’s Health Epsom United Kingdom; 7 Department of Obstetrics and Gynecology Croydon University Hospital London United Kingdom; 8 Department of Surgical Sciences, Complex Operative Unit of Gynecology Fondazione PTV Policlinico Tor Vergata, University Hospital Rome Italy; 9 Department of Obstetrics and Gynecology Universitatea de Medicina si Farmacie Iuliu Hatieganu din Cluj-Napoca Cluj-Napoca Romania; 10 Department of Obstetrics and Gynecology Faculty of Medicine Ramathibodi Hospital, Mahidol University Bangkok Thailand; 11 Department of Obstetrics and Gynecology Universitatea de Medicina si Farmacie Carol Davila din Bucuresti Bukarest Romania; 12 Urogynecology Division Department of Obstetrics and Gynecology Hospital das Clinicas da Faculdade de Medicina da Universidade de Sao Paulo Sao Paulo Brazil; 13 Department of Gynecology University Hospital Zurich University of Zurich Zurich Switzerland

**Keywords:** core outcome sets, core outcome measures sets, cosmetic gynecological surgery, intervention, labiaplasty, vulva, gynecology, cosmetic surgery, surgery, framework, outcome, effective, implementation

## Abstract

**Background:**

Studies evaluating cosmetic gynecological interventions have followed variable methodology and reported a diversity of outcomes. Such variations limit the comparability of studies and the value of research-based evidence. The development of core outcome sets (COS) and core outcome measures sets (COMS) would help address these issues, ensuring a minimum of outcomes important to all stakeholders, primarily women requesting or having experienced cosmetic gynecological interventions.

**Objective:**

This protocol describes the methods used in developing a COS and COMS for cosmetic gynecological interventions.

**Methods:**

An international steering group within CHORUS, including health care professionals, researchers, and women with experience in cosmetic gynecological interventions from 4 continents, will guide the development of COS and COMS. Potential outcome measures and outcomes will be identified through comprehensive literature reviews. These potential COS and COMS will be entered into an international, multi-perspective web-based Delphi survey where Delphi participants judge which domains will be core. A priori thresholds for consensus will get established before each Delphi round. The Delphi survey results will be evaluated quantitatively and qualitatively in subsequent stakeholder group consensus meetings in the process of establishing “core” outcomes.

**Results:**

Dissemination and implementation of the resulting COS and COMS within an international context will be promoted and reviewed.

**Conclusions:**

This protocol presents the steps in developing a COS and COMS for cosmetic gynecological interventions. Embedding the COS and COMS for cosmetic gynecological interventions within future clinical trials, systematic reviews, and practice guidelines could contribute to enhancing the value of research and improving overall patient care.

**Trial Registration:**

Core Outcome Measures in Effectiveness Trials (COMET) 1592; https://tinyurl.com/n8faysuh

**International Registered Report Identifier (IRRID):**

PRR1-10.2196/28032

## Introduction

Cosmetic gynecology is a rapidly developing area of gynecology, often closely linked to the clinical practice on the lower genital tract and relevant to the scope of practice of pelvic floor medicine and surgery, urogynecology, or plastic surgery [1].

Cosmetic gynecological interventions are elective surgical procedures aiming to “enhance the aesthetic appearance of the female external genitalia, modify genital organs, or functional vaginal procedures (in the absence of anatomic pathology), to help improve a woman’s quality of life” [2].

According to the Cosmetic Surgery National Data Bank, cosmetic female genital surgery is the second fastest growing surgical procedure domain, with an increase of over 50% in 5 years [3].

Different approaches include surgical, nonsurgical, regenerative, and energy-based techniques. Increases in labiaplasty and other labial and vulvar procedures in recent years in different parts of the world, both for reconstructive as well as for cosmetic indications, may be partly due to an increasing popularity, lifestyle, and media interest [4,5]. When the outcomes of these interventions are suboptimal or even catastrophic, lifelong implications on women’s anatomy, pelvic floor function, psychosexual function, and overall quality of life may require additional and long-term health care resources and support. On the other hand, associations between body dysmorphic disorders and requests for cosmetic procedures are well documented [6], and patient treatment pathways require robust scientific justification.

The combination of cultural shifting on body stereotypes and the patient’s specific view about vulvar anatomy that intervenes in the sexual sphere up to associations with body dysmorphic disorders makes the management and fulfilling of patient expectations in cosmetic gynecological interventions delicate and critical [6-9].

In studies on cosmetic gynecological interventions, several issues around quality of evidence are obvious [10]: research evidence is limited, and the few published studies are mainly retrospective and observational, with small numbers of patients and short-term follow up [11,12]. Validated measurement instruments are of paramount importance in this type of research as well as clinical practice, in order to support our understanding of the role and appropriateness of cosmetic gynecological interventions. However, recent research has shown that measurement instruments in published studies are highly variable, ranging from only 2 validated questionnaires for cosmetic gynecological appearance and surgery (Genital Appearance Satisfaction scale and the Cosmetic Procedure Screening Scale-Labiaplasty) to mostly no outcome measures that fulfil actual subjective or objective standards [2,13].

Cosmetic procedures can be requested by patients with functional or psychological or psychiatric disorders [4], and underlying pathologies should be assessed and managed prior to considering embarking on surgery. The analysis by Crouch et al [14] revealed that women seeking surgery had the same normal-sized labia minora similar to women in the control group not desiring cosmetic gynecological surgery. A combination of shared decision-making and questionnaires evaluating patient expectations might be a favorable approach [15].

Furthermore, psychological well-being and disorders are very difficult to assess and may greatly change with time, so that regret and revisions surgery rates should be reviewed and evaluated over sufficiently long-term follow-up periods [9,16].

The collection and reporting of outcomes and the selection of outcome measures not only in this field but also in various other areas of pelvic floor research has been largely overlooked. The consequence of this is a variety of differing outcomes that make analysis and conclusion drawing across groups of studies through systematic reviews and meta-analyses difficult, and sometimes, impossible.

A COS might increase reporting of important outcomes, reduce the risk of selective outcome–reporting, and increase the feasibility of conducting systematic reviews and meta-analyses [17].

We have developed CHORUS, an International Collaboration for Harmonising Outcomes, Research, and Standards in Urogynecology and Women’s Health (https://i-chorus.org/) with representatives from different geographical areas and academic institutions. Specific projects undertaken by CHORUS aim to tackle such limitations in research evidence and current standards. Among other initiatives, a number of systematic reviews on surgical interventions for anterior compartment vaginal prolapse, synthetic mesh procedures for the surgical treatment of pelvic organ prolapse, childbirth trauma, posterior and apical prolapse surgery, stress incontinence, and chronic pelvic pain have already been completed and published [18-25].

Addressing the variation in treatment protocols, outcome selection, and reporting represents a priority. Aiming to work on the basis of the paradigm of the Outcome Measures in Rheumatology (OMERACT) initiative could help address these issues [26,27].

This project has been prospectively registered with the Core Outcome Measures in Effectiveness Trials (COMET) initiative [28-30] (registration number 1592, Protocol version 1, registration date July 2020) [31]. The Core Outcomes in Women’s Health (CROWN) initiative will support the dissemination and implementation of a COS and COMS for cosmetic gynecological interventions to increase the value of primary research, by encouraging future POP trials to report core outcomes and thus contribute high-quality data to future meta-analyses [32].

Guidance for clinicians on how best to care for the healthy woman seeking cosmetic surgery is highly needed. The development of a COS and COMS by a multi-stakeholder group, would help address and guide patient expectations around various treatments and encourage physical and psychological long-term follow-up. COSs are minimum data sets of well-defined, discriminatory, and feasible outcomes that can be measured in a standardized manner and consistently reported [32].

## Methods

### Methods Overview

Our methodology for the development of the COS and COMS in cosmetic gynecological interventions will be based on the standards set out by the COS-STAD (Core Outcome Set-Standards for Development) recommendations [33-35]. The COS-STAD recommendations suggest that the development of a protocol at the start of the COS development process is advisable, as it increases transparency of the process.

The protocol is also in line with the COMET Initiative Handbook guidelines and other COS development research relevant to women’s health, including preeclampsia, endometriosis, stress incontinence, and childbirth trauma [34-37].

### Identifying Potential Outcomes

Selection of appropriate outcomes is an essential step of the study design. Clinical studies that evaluate cosmetic gynecological interventions must select outcomes of relevance to key stakeholders and measure them using appropriate instruments.

Mapping all outcomes reported in clinical studies evaluating cosmetic gynecological interventions in women will provide the basis for initializing the process of development of COS. This process is in accordance with the Standard Protocol Items: Recommendations for Interventional Trials (SPIRIT) statement, supported by funders of health research [38]. Data from a new systematic review that is under preparation will be the main basis of our report as none are available on patient outcomes.

In line with our previous work, a list of potential core domains on the variation in outcomes and outcome measures in cosmetic gynecological interventions from randomized trials [18,19,21] and other published research, in order to create a comprehensive inventory of potential outcomes on the basis of literature reviews and group discussions will be drafted. These inventories will form the basis for consideration of potentially eligible outcomes, and outcome measures and will be enriched during focus group consultations and the Delphi process and feedback. The COS will also comprise adverse events (AEs) and contextual factors that are reported in surgical trials. As cosmetic gynecological interventions are not attributable to a recognizable pathology (eg. genital malformation, dermatoses, or acquired disease), the identification of potential outcomes is of utmost importance. The International Society of Aesthetic Plastic Surgery (ISAPS) and the International Urogynecological Association (IUGA) are not prescriptive for the reporting of core outcomes in clinical trials on cosmetic gynecological interventions.

In the second line, data from expert guidelines (EG) and stakeholder opinions (SO) will be included and described in the *Creating an International Group for the Development of a COS for Cosmetic Gynecological Interventions* subsection.

All outcomes and outcome measures reported within the reports will be identified and categorized. Following the steering group’s agreement, the outcomes and definitions will be entered into a modified Delphi method.

The first step will be to group different definitions together under the same outcome name. The next step will be to group these outcomes into outcome domains to classify broad aspects of the effects of interventions. Categorization of each outcome definition to an outcome name and of each outcome to an outcome domain will be performed independently by 2 researchers with diverse multi-professional backgrounds.

**Figure 1:**
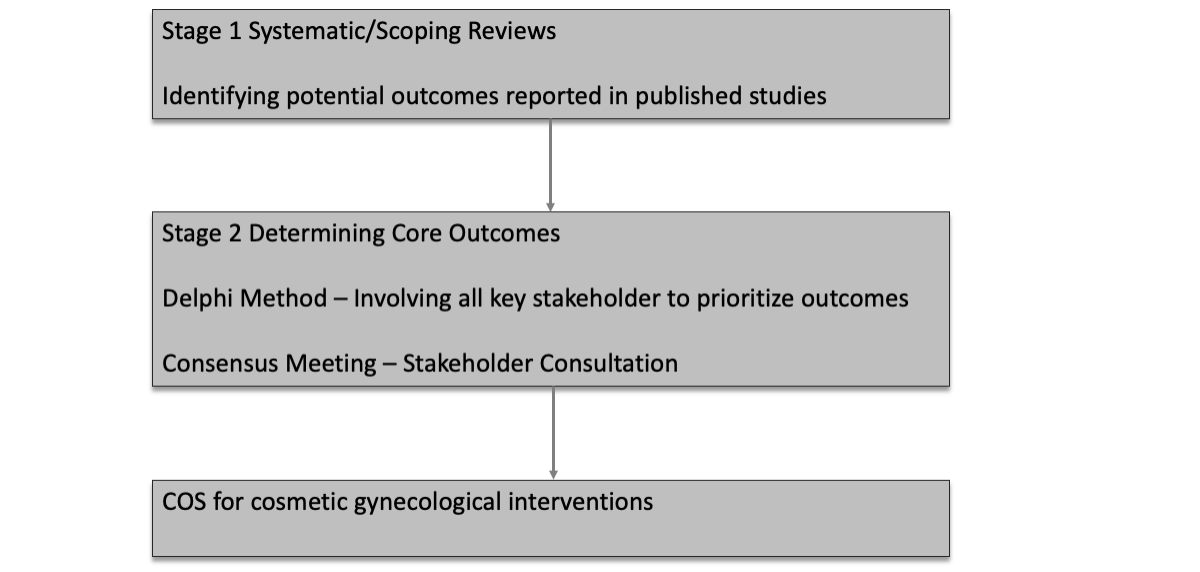
The stages of developing a core outcome set for cosmetic gynecological interventions. COS: core outcome set.

One way to overcome inconsistent selection, measurement, and divergent reporting of outcomes, is a process (Figure 1) recommended by the COMET Initiative [39]. This process has been successfully applied in several disciplines to develop, disseminate, and implement COS in cosmetic gynecology.

### Outcome Inventory

A comprehensive inventory of outcomes, identified by the systematic reviews and analyses of qualitative interviews, as described above, will be developed on the basis of standard methodology we followed in other areas of pelvic floor disorders [40]. Outcome domains will be listed in a database and coded in accordance with the taxonomy proposed by the COMET Initiative.

If there is uncertainty regarding how to classify or present an outcome, consensus of the steering group will be sought. Following the steering group’s agreement, the outcome inventory will be entered into the modified Delphi method.

### Creating an International Group for the Development of a COS for Cosmetic Gynecological Interventions

An international steering group within CHORUS, including health care professionals from different geographical areas and disciplines; key opinion leaders such as plastic surgeons, urogynecologists, gynecologists, colorectal surgeons, urologists, general practitioners, researchers, policy makers, industry representatives, and professional societies’ representatives; and most importantly women with experience of cosmetic gynecological interventions will lead the development of these COS and COMS [41]. In line with methods developed and endorsed by COMET (reference COMET Handbook), key stakeholders performing vulval surgery (self-declaration) or publishing on cosmetic gynecological interventions (randomized clinical trials or systematic reviews that are published in internationally peer-reviewed journals) will be identified, approached, and selected for representativeness via professional societies (ISAPS and IUGA) and the CHORUS website (i-chorus.org and social media). There are no clear recommendations for calculating the required sample [42]; based on previous studies, we will aim to include a minimum of 20 participants from each stakeholder group.

### Generation of a List of Potential Core Domains

Potential core outcome domains to be selected and evaluated will comprise patient satisfaction and surgical outcomes and may further include morbidity, quality of life and life impact, resource use and economic impact, pathophysiological and psychological manifestations, choices influence by context, long-term impact, and adverse events [43]. A specific health framework on definitions and domains in cosmetic gynecological medicine is not available yet. The collated COS will not be categorized into primary or secondary outcomes as the prioritization will be designated by future studies. A COS will not preclude measurement of additional outcomes if relevant to a specific trial. Recommendations about the weighting of COS domains, however, will be proposed depending on the data from systematic reviews (SR), EGs, and SO. The category of recommendation will be declared (SR, EG, and SO). These COS and COMS will be applicable to clinical studies evaluating therapeutic interventions for women with cosmetic gynecological procedures.

This group will address the need for the development of effective interventions to improve the outcomes of cosmetic gynecological procedures and the effect these procedures may have on women’s quality of life, considering the flaws and weaknesses of current evidence. Updates will be provided on a biyearly basis.

### Study Management

The project for developing a COS and COMS for cosmetic gynecological interventions will be supervised by a management team and a steering committee. The management team will meet monthly and organize day-to-day tasks and overall work progress. The management team will include research partners. The steering committee will meet at 6-month intervals and will include an independent chairperson and 2 other independent expert members who can provide advice on methodology and cosmetic gynecology–related issues. There will also be representatives from the management team. The role of the steering committee will be to provide support and guidance. The designed CHORUS members, from different geographical locations, will directly identify patients or will be helped to contact patients by other health care providers who could have direct contact to patients. Women with experience in cosmetic gynecological interventions will be invited to participate in the study management and oversight process after sending them an informative document.

The question regarding ethics has been considered and addressed in a number of previous COS development projects. It has been suggested that these projects are considered a service evaluation not directly influencing patient care or safety [26,34,35,37,44]. Consent will be sought from all participants involved before their participation in either stakeholder interviews or the Delphi survey. All procedures will be conducted in accordance with the tenets of the Declaration of Helsinki. A “no-response” option will be allowed both for the survey and interactive parts of the study, to ensure responders’ right to withhold information. A specific timeframe of the Delphi process will be provided and information concerning the interval of data storage and handling will be made available to all participants.

### Modified Delphi Method

The core outcomes will be determined using a modified Delphi method [45], where several surveys are delivered over a series of rounds. The Delphi method consists of sequential web-based surveys that constitute consecutive rounds in an anonymous way. After each round, the group responses are fed back to the respondents who can reconsider their views on the basis of the report of the group views [43]. The modified Delphi method has advantages over less structured consensus methods. Web-based Delphi surveys facilitate international participation and are considered feasible, efficient, and acceptable to the user [46,47].

#### Round 1

Participants will be asked to register on the internet, provide demographic details, and commit to all 3 rounds. They will be allocated a unique identifier, which will anonymize their responses.

Delphi survey round 1 will contain a list of outcomes to be scored, ordered alphabetically by domains. The list of outcomes will include the option to display a more detailed plain-language description. Participants will be asked to score individual outcomes, using a 7-point Likert Scale, anchored between 1 (not important) and 7 (critical). This scale was created by the Grading of Recommendations Assessment, Development and Evaluation (GRADE) working group, and it has been widely adopted by COS developers [48]. There will be provision for an option for participants to suggest additional outcomes.

For each outcome, the median (IQR) values of scores will be calculated and summarized graphically for the whole and individual stakeholder group responses, using GraphPad Prism (GraphPad Software). Additional outcomes listed by participants will be reviewed by the outcome committee and, if novel, listed in round 2. The round will close following a 4-week window.

The number of participants in each stakeholder group who respond to round 1 will be assessed at the end of the round. Results will be presented as n (%) values for the following parameters: (1) registrations, (2) respondents who have completed the survey, (3) respondents who completed the round, (4) respondents in each stakeholder group, (5) respondents compared to potential respondents, as identified from the information provided by clinical leads, and (6) new respondents who were not included in original invitation to complete the survey.

#### Round 2

Participants will be informed regarding the outcome scores from the previous round. After revealing their own score, participants will be invited to rescore each outcome. Any changes in the score from round to round will be noted. The round will close following a 4-week window.

The modified Delphi method encourages repeated reflection and rescoring, promoting whole and stakeholder group convergence upon consensus “core” outcomes [49]. These rounds’ results will enable individual outcomes to be classified as shown in Table 1 [42]. These definitions and criteria have been proposed by previous COS developers [17].

The feedback report of the second round will be presented to all participants invited. A priori consensus will be set at 67% of the panel agreeing that a domain will be core, with domains reaching this threshold to be included in this COS. If there are clear discrepancies between stakeholder groups or if controversial arguments emerge, the results will be presented to the Steering Committee for final decisions. Examples exist where patients identified an outcome important to them as a group that might not have been considered by clinicians on their own [17]. Controversial arguments will be reported in the COS and COMS publication on cosmetic gynecological interventions. Round 2’s results will also be reviewed by the steering group to consider whether a further Delphi survey round (round 3) is required.

**Table 1:** Consensus status based on core outcome criteria.

Consensus status	Description	Criteria
Consensus in	Classify as a core outcome	Over 70% of participants in each stakeholder group score this outcome domain “critical” and less than 15% of participants in each stakeholder group score the outcome domain “not important.”
Consensus out	Do not classify as a core outcome	Over 70% of participants in each stakeholder group score the outcome domain “not important” and less than 15% of participants in each stakeholder group score the outcome domain “critical.”
No consensus	Do not classify as a core outcome	Anything else

### Presentation of the COS/COMS

The analyses will be primarily descriptive, with frequency counts provided for the variables. A limited number of analyses for trends within categorical variables (chi-square or Fisher’s exact test) will be performed. These analyses examine the relationship between measures of consensus, the different stakeholders, and diagnostic criteria.

### Stakeholder Consultation

During this final phase, a consultation chaired by an independent coordinator will be undertaken, with the purpose of selecting outcomes to be validated and included in the COS. In addition, outcomes that do not meet core outcome criteria will be discussed and considered. This consultation will purposefully include various points of view from participants who have completed all rounds of the Delphi survey. During the consensus process, the results from each round of the Delphi survey will be presented. To avoid biased consensus formation among a group of varied participants, the steering committee will consider all opinions [32] in an interactive consultation. To facilitate dissemination and implementation, editors from key journals and funders of cosmetic gynecological research will be invited to participate.

## Results

CHORUS aims to develop and sustain a robust research culture and clinical excellence by promoting, conducting, and implementing research that not only contributes to improvements in the knowledge base and patient care, but also informs the development of clinical standards and aims to improve clinical services for patients and users. With the implementations of COS and COMS, comparability of primary research and findings of meta-analyses and systematic reviews will become more scientifically sound and clinically relevant. Commitment to delivering a research agenda that is focused on enhancing clinical and cost-effectiveness and on systematic measures to monitor and improve quality will add value to research and its purpose to inform clinical practice. Funding bodies will not have any involvement in the design, conduction, analysis, and interpretation of data, or in writing the manuscript and the publication process.

## Discussion

The efficacy and safety of cosmetic genital procedures, scientific justification, and validation should be confirmed through rigorous evaluation. Meta-analyses based on high-quality trials are highly warranted. The development of core outcome sets (COS) and outcome measures sets (COMS) will form the basis for better quality research to inform clinical practice and support patient-centered care.

As dissemination is the pivotal step in the effective application of trial outcomes, this will be planned in detail, drawing on the necessary expertise, at the outset of any research undertaking. The full report and academic publication will report with reference to the COS–Standards for Reporting statement and checklist [33].

The National Institute for Health and Care Excellence recommends the use of COS when selecting outcomes during evidence scoping and synthesis [39]. As the output of this activity may form the basis of developing guideline recommendations, the COS and COMS could have a direct impact in improving health care for women undergoing cosmetic gynecological interventions. Consensus on both domains and instruments achieved by interdisciplinary groups of different relevant stakeholders, including patients, will improve future health care for women and should be implemented in medical education.

## Abbreviations

AE: adverse event
CHORUS: An International Collaboration for Harmonising Outcomes, Research and Standards in Urogynecology and Women’s Health
COMET: Core Outcome Measures in Effectiveness Trials
COMS: Core Outcome Measure Set
COS: Core Outcome Set
COS-STAD: Core Outcome Set-Standards for Development
CROWN: Core Outcomes in Women’s Health
EG: expert guidelines
GRADE: Grading of Recommendations Assessment, Development and Evaluation
ISAPS: International Society of Aesthetic Plastic Surgery
IUGA: International Urogynecological Association
SO: stakeholder opinions
SPIRIT: Standard Protocol Items: Recommendations for Interventional Trials
SR: systematic review
